# National commitments to Aichi Targets and their implications for monitoring the Kunming-Montreal Global Biodiversity Framework

**DOI:** 10.1038/s44185-024-00039-5

**Published:** 2024-04-03

**Authors:** Calum Maney, Daniela Guaras, Jerry Harrison, Alejandro Guizar-Coutiño, Michael B. J. Harfoot, Samantha L. L. Hill, Neil D. Burgess, William Sutherland

**Affiliations:** 1 United Nations Environment Programme World Conservation Monitoring Centre (UNEP-WCMC), 219 Huntingdon Road, Cambridge, UK; 2 Vizzuality, 123 Calle de Fuencarral, 28010 Madrid, Spain; 3 Conservation Science Group, Department of Zoology, University of Cambridge, Cambridge, UK

**Keywords:** Biodiversity, Environmental impact, Policy

## Abstract

The Convention on Biological Biodiversity (CBD) exists as a major multilateral environmental agreement to safeguard biodiversity and “live in harmony with nature”. To deliver it, strategies and frameworks are set out in regular agreements that are then implemented at the national scale. However, we are not on track to achieve overall goals, and frameworks so far have not been successful. This could be due to unambitious targets, low follow-through on commitments, or desired outcomes for nature not being achieved when action is taken. Here, we focus on national planning and reporting documents from a set of 30% of Parties to the CBD. We found that nearly half of the commitments mentioned in national planning documents did not appear in the Sixth National Reports and that further losses emerged due to measures reported as incomplete or ineffective. There were differences between commitments to each of the Aichi Targets, with more losses in high-profile and “institutionally challenging” Targets. Commitments from Parties in different Human Development Index categories had different outcomes among Targets, and Parties self-identifying as “megadiverse countries” had overall higher rates of reported success. Our results are important for informing the monitoring of commitment implementation in the Kunming-Montreal “global biodiversity package”.

## Introduction

The Convention on Biological Diversity (CBD) is the most pivotal multilateral environmental agreement (MEA) enacted in response to concerns about biodiversity loss^1^. Adopted in 1992, the CBD had 196 Parties as of the end of 2021^2^. Following the adoption of the Strategic Plan for Biodiversity 2011–2020 and its Aichi Biodiversity Targets and its progress over the “decade of biodiversity”, Parties were encouraged to use it as an overarching framework to guide plans and implementation at the national level.

National Biodiversity Strategies and Action Plans (NBSAPs) were developed by Parties to support the delivery of the Strategic Plan and deliver the Aichi Targets. Analyses of progress towards the Aichi Targets^3,4^ showed major shortfalls, with a failure to achieve almost all targets, leading the scientific community to stress the importance of increasing global ambition^5–7^ and closing national implementation gaps^1,8–10^.

Parties to the CBD are obliged to submit National Reports on measures taken towards the implementation of the commitments in their NBSAPs, as well as the effectiveness of the measures taken in reaching targets. The most recent such reports were the Sixth National Reports, submitted by 164 Parties by January 2020. These documents report measures taken towards implementing the NBSAPs and progress towards achieving targets. Some research has analysed progress for specific targets or Parties^11–14^.

NBSAPs, by their nature, are unwieldy, idiosyncratic in format, and inaccessible to analyse—especially between Parties, as there is no standardisation in the structure. Though often itemised and laid out in consistent hierarchical structures, they are presented in unstandardised tabular formats or simply lists; only a small number of NBSAPs are available in the form of commitment databases^15^. National Reports do not necessarily reference NBSAPs directly, which often makes relating their contents to the relevant NBSAP challenging. Furthermore, for most reporting Parties, the commitments reported as having been carried out and carried out effectively do not line up directly with actions, objectives, or even targets in their NBSAPs.

Some in-depth analyses have been done by the CBD Secretariat to inform the Convention’s subsidiary body on implementation at a higher level, assessing the adoption of NBSAPs as a policy instrument with adaptations for national circumstances^16,17^. However, there has been no formal investigation of the delivery of commitments towards the Aichi Targets at a national level using information from NBSAPs and Fifth or Sixth National Reports^18^. Therefore, any potential insights from these resources in investigating the overall progress in delivering planned actions are still unknown^16,17^.

In this analysis, we assess the effectiveness of the implementation of the Aichi Targets at the national level. We use NBSAPs as a source of national commitments towards achieving the Aichi Targets and Sixth National Reports as a source of information on the implementation of those commitments. We then use an analysis of a subset of NBSAPs and Sixth National Reports to define and measure gaps in implementation over the past 10 years and use these insights to provide guidance for the implementation of the new Global Biodiversity Framework and its monitoring framework.

Parties in this subset have variable capacity to carry out biodiversity-related activities; this may impact their capacity to plan, implement, and monitor commitments to biodiversity. We use the categorised form of the human development index (HDI) to represent this in our analysis, as the HDI is a compound index reflecting elements of living standards, health outcomes and poverty^19^. Further, national relationships with and perceptions of biodiversity also differ. To investigate how these interact with engagement with commitments under the CBD, we also compare overall progress between Parties within and outside of the group of self-identified “Like-minded megadiverse countries” (LMMCs)^20^, hypothesising that Parties within this group give greater precedents to biodiversity issues, and thus may be more effective in implementing their commitments. The LMMCs are a group of self-determined “megadiverse” countries, together harbouring over half the world’s threatened species as listed by the International Union for Conservation of Nature^21^.

Our results have implications beyond the Aichi Targets and the 2011–2020 period. A new agreement under the CBD was made in December 2022 to follow on from the Aichi Targets, with targets agreed for 2030 and 2050. This “Kunming-Montreal Global Biodiversity Framework” represents the newest phase of agreements under the CBD, with a pledge to implement a monitoring framework to measure progress (these two elements are two of the five areas of work that comprise the Kunming-Montreal “global biodiversity package”)^22^. The overall package builds on the experience of previous cycles, both within and beyond the scope of national interventions, as it aims for a broader and more holistic implementation approach, including the private sector, setting more ambitious targets, and increasing available funding. It comes after a long negotiation targeted at combatting perceived weaknesses in the Aichi Targets, with disagreement among blocs with varying priorities and needs. With only seven years left to achieve the new goals, understanding how to make the many commitments already made effective is a priority^23^. A novel part of the new Kunming-Montreal “global biodiversity package” is the proposed use of standard-form reporting in National Reports, such that transparency in global progress assessments is increased. This means an assessment of the informational value of such existing documents is valuable.

## Results

### Initial analysis and overall patterns across Targets

Here, using a combination of text-mining techniques and manual extraction, we created two databases of planned (NBSAPs) and implemented (Sixth National Reports) actions. We joined these datasets together to allow for comparison between Parties that have reported variable coverage on their successes. We took a subset of Parties’ national planning and reporting data that were available from the CBD Clearing-House Mechanism with a temporal cut-off of 31st January 2020. Then three criteria were applied to select Parties: (1) Parties needed a Sixth National Report and a connected NBSAP (this narrowed the subset to 90 Parties); (2) Parties that have submitted a Sixth National Report to also have submitted an NBSAP in the period after the Aichi Targets were adopted, 2011-onwards (64 Parties met criteria 1 and 2); (3) Parties had itemised, hierarchical action plans in their NBSAPs. Our final subset comprised 58 Parties, covering a range of regions and Human Development Index (HDI) categories (Supplementary Fig. 1, Supplementary Table 1).

**Fig. 1:**
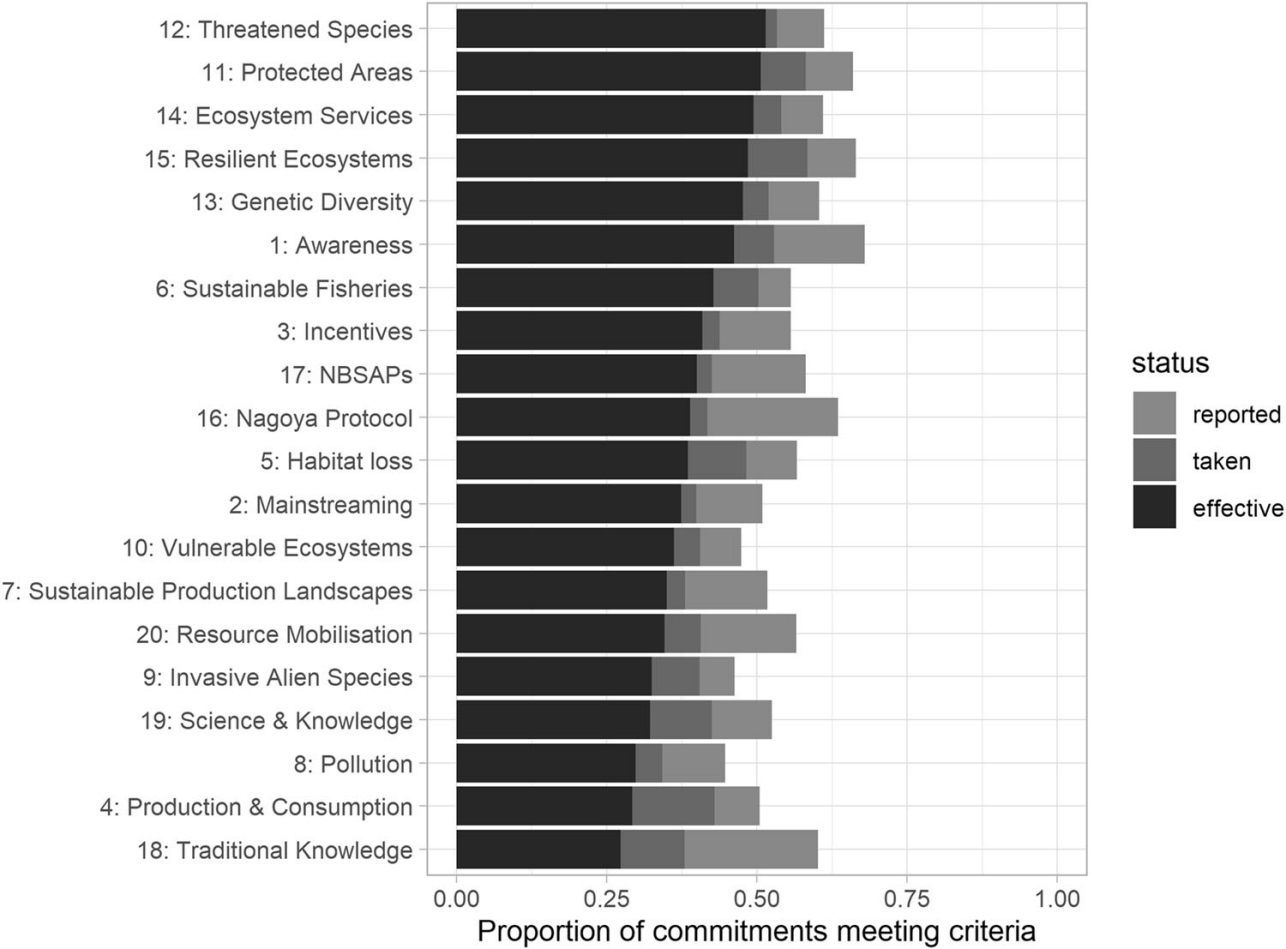
An assessment of the proportion of commitments from NBSAPs (National Biodiversity Strategies and Action Plans) that are referenced by Sixth National Reports. 1.00 represents the full proportion of commitments made in the studied NBSAPs linked to each Aichi Target, and each colour block represents commitments which have increasingly positive and detailed reporting. The black bars represent the proportion of commitments towards each Aichi Target which were reportedly carried out effectively by Parties.

A total of 7931 commitments were identified as planned in all 58 NBSAPs. About half (4425) were mentioned with a measure corresponding to them in the relevant Sixth National Report (in results: *reported*). Of these 4425 commitments, 3617 were determined to have been implemented to some degree in National Reports (labelled in our results as ‘*taken’*), while 3221 were classified as at least partially effective (‘*effective’*)—which here reflects where a commitment to biodiversity made in a Party’s NBSAP was referenced in the Sixth National Report, and where the level of progress recorded at the relevant reporting level was either “partially effective” or “effective” in the self-assessment. The Commitments most highly represented in “effective” measures are those linked to Aichi Targets 11 (Protected Areas) and 12 (Threatened Species), whereas those least classified as “effectively implemented” were those linked to Targets 8 (Pollution), 4 (Production and Consumption), and 18 (Traditional Knowledge) (Fig. 1).

### Patterns among groups of Parties

**Fig. 2:**
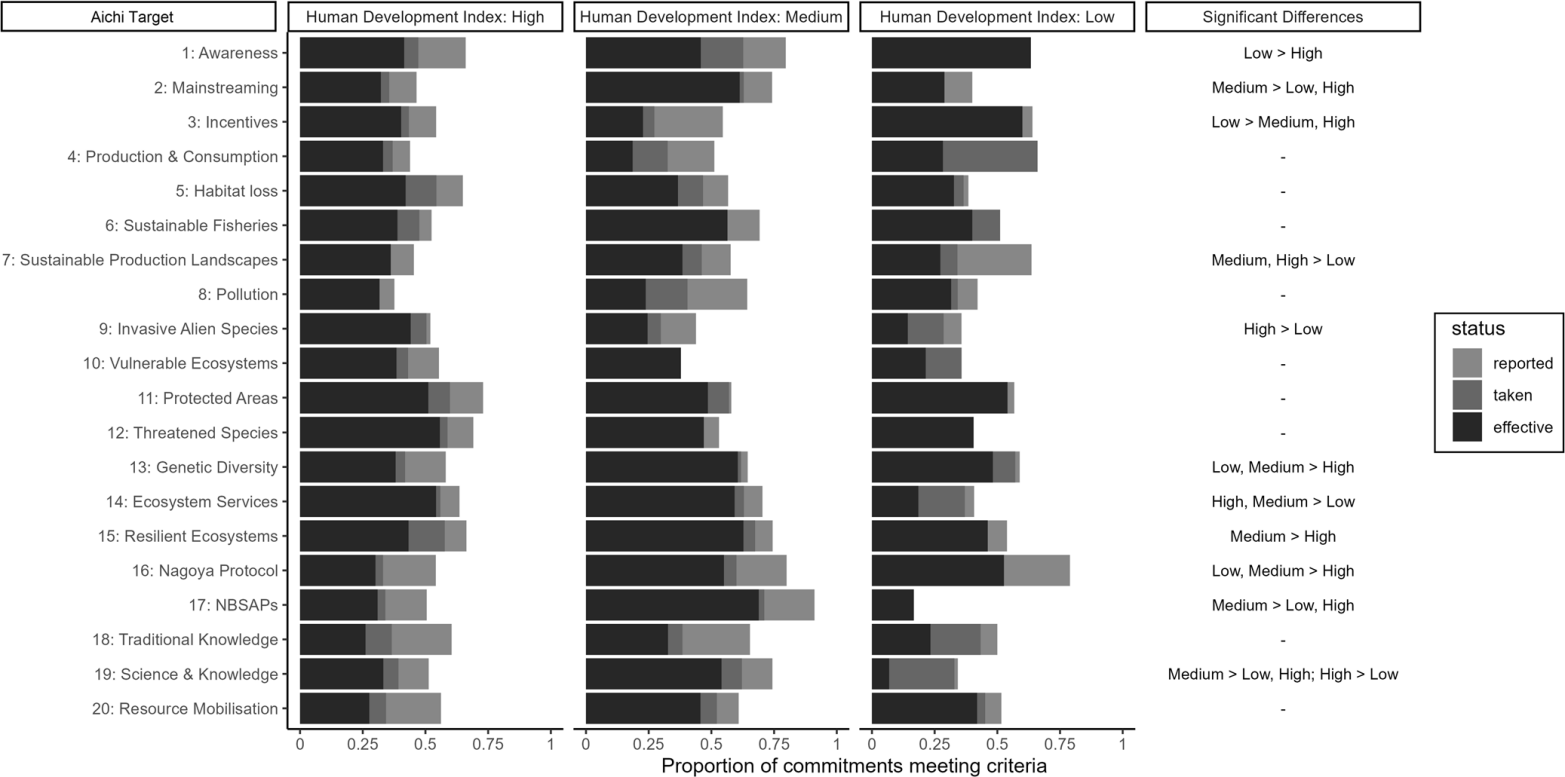
National implementation of the Aichi targets disaggregated by Human Development Index (HDI) category. Labels represent where a significant difference was detected between commitments made in each category. On the right, differences among the three HDI groups are displayed, showing where there were significant differences in the proportion of commitments reported as “effective” between Parties in the different groups.

There was no consistent discernible effect, as shown by logistic regression, of the Human Development Index (HDI) category of the Party that made a commitment on the reported delivery of that commitment when the analysis was ambivalent to any links to Aichi Targets. However, the likelihood of a commitment to be recorded as effective did differ when the results were divided by Aichi Target (Fig. 2). For example, a smaller proportion of commitments made by Parties in the low HDI category related to Aichi Target 7 (Sustainable Production Landscapes), Target 9 (Invasive Alien Species), and Target 14 (Ecosystem Services) were reported as effective. However, in other areas, such as Target 1 (Awareness), Target 3 (Incentives), and Target 16 (Nagoya Protocol), commitments made by Parties in the low HDI category appeared to be more likely to be reported as effective. Moreover, it was commitments made by Parties in the Medium HDI category which, in 8 of the Targets, higher reported successes in implementations than at least one other group. Only for commitments towards Target 19 (Science and Knowledge) was there a clear hierarchy in reported effectiveness, with the Medium group higher than both others and the High group higher than the Low group.

**Fig. 3:**
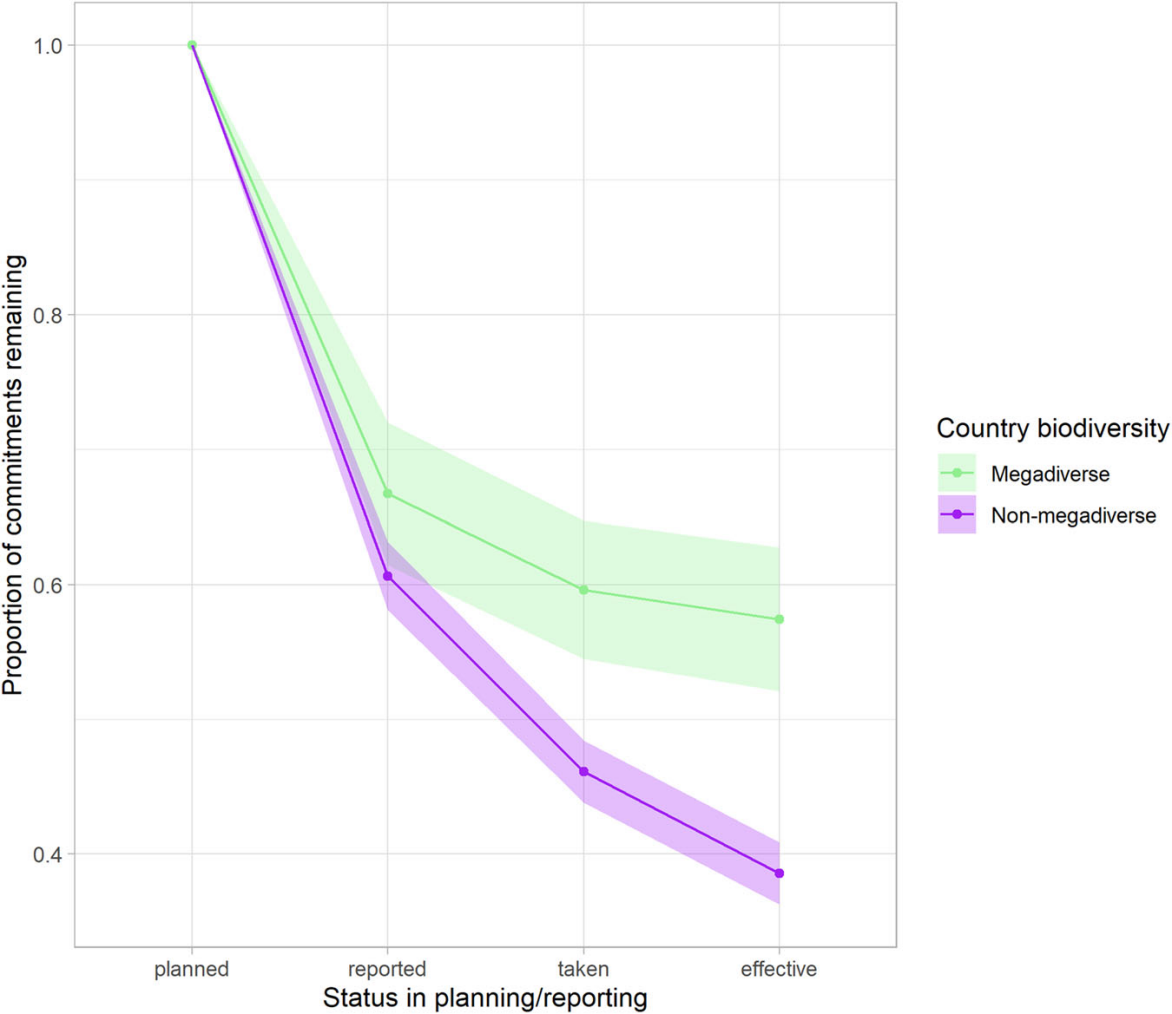
Proportional decay in commitments made in NBSAPs by megadiverse and non-megadiverse countries. Bands represent 95% confidence intervals over Parties in and out of the subset. Commitment progress categories are described in Fig. 1.

Parties of the “Like-Minded Megadiverse Countries”^20^ experienced a similar level of “omission” to non-megadiverse countries (though this was slightly lower in the LMMCs). However, national reporting suggests that reporting on Parties from megadiverse countries was more consistently taken and effectively implemented (*p* < 0.001*, F* = 23.6) (Fig. 3).

## Discussion

For the past Strategic Plan, Sixth National Reports showed effective delivery of fewer than half of the contents of our sample of 58 NBSAPs. Furthermore, across our sample of CBD Parties, there was a marked decline in the number of commitments along each stage of the analysis: planned, action-reported and implemented. Moreover, a large proportion was not reported: 44% of planned activities with a completion date before 2020 did not appear in National Reports. Further, some actions that did appear in National Reports came with a report that no steps had been taken towards achieving them. Finally, not all actions undertaken were reported as effective by the Parties. This evidence, alongside other evidence that international ambition for biodiversity conservation has been too low^24,25^, and the insufficient capacity to deliver on NBSAPs^26^, helps explain why the Aichi Targets were not met^4^.

Where there is little evidence for implementation successes (especially where commitments have been reported as abandoned, unfinished or ineffective), we can infer that the barriers to achieving conservation goals have impacted progress in different ways. For example, the widespread reporting of commitments towards Target 4 (Sustainable Production and Consumption) as ineffective (or their omission from National Reports) may have arisen from the relatively low governmental priority that NBSAPs hold in many countries compared to other sectors like agriculture or industry. These sectors can have considerable influence on NBSAP formulation and implementation, but the government departments responsible for NBSAPs have little or no control over them^27^. Additionally, entities in the private sector, especially transnational corporations, hold stewardship over much of the world’s resource production and its associated impacts on biodversity. Insufficient engagement of national strategies with these bodies means it may have been difficult to achieve and track many of the commitments made towards this Aichi Target^28^. Finally, it has been previously suggested (using an analysis of commitments towards the Aichi Targets) that where commitments challenge institutional norms and historic governance, they are less likely to be effectively implemented^9^. Whilst this is not necessarily the full explanation of why targets such as Target 2 (Mainstreaming), Target 18 (Traditional Knowledge), and Target 20 (Resource Mobilisation) are among those targets with proportionally few reports of effective commitment implementation, it should be noted that commitments towards such targets may require additional support and cross-sector prioritisation if they are to succeed in the future. Some research has linked overall progress towards the Aichi Targets to their “SMART” characteristics^29^: targets with more specific, measurable, ambitious, realistic, and time-bound characteristics were found to be more likely to be associated with higher levels of linked indicators. The results of this analysis are mirrored, in part, by our findings. Notably, Targets under Goal C (11, 12 and 13) were classified as having the most SMART characteristics; they were also all in the top 5 most effectively implemented Targets in this analysis. However, this was not universally the case, perhaps linked to the fact that in many national-level translations of the Aichi Targets, wordings were altered, potentially with different SMART characteristics.

This analysis provided further evidence that some Aichi Targets were harder to achieve at a national level than others, such as 9 (Invasive Alien Species) and 20 (Resource Mobilisation). For each of these, inferences can be made as to why reported national delivery was, on average, poorer. Target 9 (Invasive Alien Species) was predicated on high scientific and technical capacity both for their implementation and monitoring^30^; given the variability of this capacity across Parties, such requirements are likely to have contributed to reported variation in achievement. Target 20 (Resource Mobilisation) relied on buy-in from national and international funding sources, including national treasuries. This means that often, monetary resources were limited by shifting national priorities and international conservation interests^9^. The parts of the new Kunming-Montreal Global Biodiversity Framework that engage other sectors and stakeholders will therefore be crucial to effective monitoring and implementation.

The lower proportion of commitments related to Aichi Target 9 (Invasive Species) reported as successful by Parties in the low HDI group suggests that difficulties arose in areas where financial and technical capacity may be lower when carrying out and monitoring the effects of commitments towards this Aichi Target. Evidence suggests that this may be a particular problem for the implementation of Aichi Target 9 (Invasive Alien Species), which requires high technical and scientific capacity for effective monitoring and control of invasive alien species (IAS)^30^. Benefits could be derived from additional support in IAS-based commitments in lower-HDI Parties for both national-level achievement and overall global ambition^31^.

Broadly, commitments to Targets under Strategic Goal A (1–4), aimed at addressing the underlying causes of biodiversity loss, were reportedly better delivered by Parties in the Low and Medium HDI categories (Fig. 2). However, no group had significantly higher reported effectiveness in Target 4 (Production & Consumption), highlighting the pervasive difficulty of achieving this Target. There was also no overall pattern across Targets under Strategic Goals B and C, addressing direct drivers of biodiversity loss and biodiversity-positive commitments, respectively. Strategic Goals D and E, focusing on benefits from biodiversity and enhancing implementation of the framework, both showed an overall pattern where commitments made by Parties in the Medium HDI category were more likely to be reported as successful than commitments from other groups (Targets 14 (Ecosystem Services), 15 (Resilient Ecosystems), 16 (Nagoya Protocol), 17 (NBSAPs), and 19 (Science and Knowledge)). It could be suggested that, at least in terms of biodiversity benefits, such Parties may have achieved an adequate balance of resources to carry out commitments towards biodiversity while maintaining more direct dependencies on nature that may have been substituted in higher-HDI Parties^32^.

Targeted support for Parties based on where they are most likely to face shortfalls could have been applied across the suite of Aichi Targets. For example, low-HDI countries could have, according to the Sixth National Reports, minimised the largest shortfalls with support towards Target 8 (Pollution), 9 (Invasive Alien Species), 14 (Ecosystem Services), and 19 (Science and Knowledge). Similarly, it would appear from the Sixth National Reports that medium-HDI countries could have benefitted most from support in delivering commitments to Aichi Target 3 (Incentives), 4 (Sustainable Production and Consumption), 8 (Pollution), and 9 (Invasive Alien Species).

The group identified as “Like-Minded Megadiverse Countries” had, on average, reported the effective delivery of a higher proportion of the commitments in their NBSAPs than Parties outside this group (Fig. 3). The reported effective commitment delivery in megadiverse countries was particularly prominent when the commitments concerned certain Aichi Targets, including Targets 3 (Incentives) and 20 (Resource Mobilisation), Targets 6 (Sustainable Fisheries) and 9 (Invasive Alien Species), Target 14 (Ecosystem Services), and Targets 18 (Traditional Knowledge) and 19 (Science and Knowledge) (Supplementary Fig. 2). This suggests a particular willingness amongst the like-minded megadiverse countries to prioritise biodiversity, delivering commitments that may challenge institutional norms or require considerable prior funding, capacity, and engagement. Megadiverse countries have previously been shown to also have more consistent use of indicators in their reporting^33^. Our results lend weight to the idea that, in general, these Parties have more readily participated in the CBD and acted with greater consciousness of the importance of biodiversity and its equitable and sustainable use. However, our results show no difference in the reported success of commitments from megadiverse countries towards Aichi Targets such as 4 (Production and Consumption) and 5 (Habitat loss). This is of concern, as habitat loss and other pressures on species from productive sectors in the megadiverse countries will contribute to much greater losses in global biodiversity than the same pressures elsewhere. The engagement of other government sectors, the private sector, and environmental non-governmental organisations could help to close this gap in the future for megadiverse countries.

This analysis focuses on the Sixth National Reports, which were first submitted in 2014. This cycle of reporting does not necessarily line up exactly with the timings of the NBSAPs, as the earlier Fifth National Reports may have included some assessment of commitments omitted from the reports in this analysis. Additionally, the Sixth National Reports contained over 100 sets of measures that did not correspond to commitments in their corresponding NBSAP, and though they do not necessarily contribute to the fulfilment of commitments made in NBSAPs, they are still likely to contribute to the Aichi Targets.

The principal caveat to these findings is that they are founded on the Sixth National Reports. These documents are, to all extents and purposes, self-reported evaluations of progress. This means that there can be no direct qualification of progress made towards national and international biodiversity goals through methods using reporting documents alone unless a thorough and transparent system for evidence-based reporting can be established. Though evidence and supplementary report documents are often provided in current reporting, this is not consistent across parties or commitments within. Considering this, we do not draw direct comparisons between individual Parties in this analysis.

The subset of Parties in this study may also have affected the spread of results. As our methods exclude Parties which have not precisely reported the outcomes of their objectives, this may introduce a bias towards certain Parties. Geographically, there are relatively few Parties in the Western Europe category, as few of these Parties met the criteria for inclusion and were also represented by the wider EU plan. However, the geographical spread of the other assessed Parties was more representative. Africa and ‘Asia and the Pacific’ were the largest groups, followed by ‘Latin America and the Caribbean’.

Further, the focus of this analysis on single commitments as the unit of measurement could lead to misinterpretation of Parties’ achievements towards the Aichi Targets. It is possible that, even with a small proportion of commitments effectively implemented, the most important and ambitious commitments were still completed. Without a way to properly measure the importance of every commitment, it is important again to stress that the methods here should not and do not address the progress of any one Party towards achieving the Aichi Targets.

Though this study is correlative, and the potential of confounding effects cannot be excluded, associations of effective commitment delivery with economic capacity and biodiversity exist over a large number of Parties. From this, we can reasonably infer the existence of barriers to progress that differed in different countries when addressing various types of goals.

By 2020, national commitments toward the Aichi Targets had not led to the achievement of the original goals^4^. Still, at the commencement of negotiations on the Kunming-Montreal Global Biodiversity Framework, hundreds of new pledges were already being made under the Action Agenda^23^; work on revising new NBSAPs and reporting documents is also underway. With the Kunming-Montreal Global Biodiversity Framework comes the Monitoring Framework, linking the outcomes of the framework to measurable indicators^34^. Considering how many commitments fell out of the reporting framework in this analysis and how patterns among target themes emerged, it is important to carry forward lessons from the Aichi Targets when considering how to get the most out of this framework and make it effective in achieving the new Goals and Targets.

Our analysis shows fewer and fewer actions taken at each stage of the implementation process of the last strategic plan for biodiversity. Some of this, as we hoped to investigate, is linked to commitments that weren’t successfully implemented, were cancelled, or otherwise implemented without their desired impact. However, the bulk of “lost” commitments were simply not reported on. This may be due to unwillingness to report on failed objectives or simply the fact that the evidence for those commitments was not available. However, this shortfall in reporting has clear implications for new plans for monitoring commitments. Compiling National Reports with this current coverage of commitments into a global progress report, as planned for the Kunming-Montreal Global Biodiversity Framework’s monitoring framework^34^, would provide an inadequate and flawed perception of the implementation and success of commitments.

A recent perspective highlighted several key steps that may be necessary to enhance the national and subnational implementation of the new global biodiversity targets^35^. One challenge they identified was ‘imperfect review mechanisms’; they suggested that one way to improve this would be a new compliance and accountability mechanism. For the monitoring framework to succeed, the coverage of commitments in each National Report should increase urgently. We suggest that a full review of specific Parties’ contributions to international targets, from commitment-setting to implementation monitoring, in the style of the analysis presented here, should be a key part of this process. Knowing explicitly about failures as well as successes and the specific conditions that led to them will be invaluable for increasing the effectiveness of the Kunming-Montreal Global Biodiversity Framework. In particular, doing this consistently could provide an evidence base with which Parties could learn from one another, co-developing knowledge of “what works” in national biodiversity policy. Further, where systematic issues with implementation arise, such a system could help identify pathways to resolving them. Even under the current system, we suggest missing commitments be counted as unimplemented in the compilation of National Reports to avoid any misrepresentation of missing commitments, providing an overly optimistic view of implementation progress.

The Kunming-Montreal Global Biodiversity Framework aims for a *“Global review of collective progress in the implementation of the Kunming-Montreal global biodiversity framework including the means of implementation, based on national reports and, as appropriate, other sources”*^22^. In our observations of commitments’ successes and failures in the National Reports, we see the compilation of National Report data as one possible route to such an analysis. However, for this to comprehensively capture the implementation of national commitments to biodiversity, direct links between clear commitments and the reporting of those commitments are needed. When paired with new indicator data, comprehensive reporting could also aid an understanding of how implemented commitments can lead to a true improvement in the state and trends of biodiversity, providing better inference for what actions work well in achieving biodiversity goals.

Our work with NBSAPs and reporting to the last strategic plan for biodiversity provides considerable insights for the implementation of the new GBF and its monitoring framework. For example, disaggregating the steps between setting national targets and measuring changes in national-level indicators added to our understanding of where and how shortfalls in commitment-making and implementation may have prevented the achievement of the Aichi Targets. As such, an enhanced monitoring system, such as the one planned for the new Global Biodiversity Framework, using harmonised planning and reporting data that brings in multiple lines of input data, could enhance transparency and accountability in the national implementation of commitments to biodiversity. It could also enable targeted interventions from Parties to prevent certain national targets and pledged commitments from being “left behind” as shown in this analysis.

If individual commitments are clear, and reporting against each of these commitments is consistent, it is possible to identify where progress has been made and where challenges exist in the national implementation of global biodiversity targets. An effective monitoring process could combine consistent reporting of commitments (such as in this analysis) with assessments of how delivering commitments contributes to the achievement of biodiversity goals, for example, by scaling commitments to impacts through a Global Biodiversity Observation System linked to a detection and attribution framework^36^. This has been done for some Parties such as Germany^12^, and indicator-led analyses of the impact of national commitments are partly available across all target elements and Parties^18^. Additionally, a consistent and transparent reporting procedure could allow for fairer comparison between Parties, as well as provide a workable estimate of the extent to which national action plans are being carried out^10,35^. Finally, if the new Global Biodiversity Framework can improve reporting transparency and accountability against targets and indicators, this will significantly help efforts to achieve the CBD 2050 vision of living in harmony with nature.

## Methods

In this analysis, we expected that across a suite of Parties and stages of implementation, shortfalls would be found at every stage, though these will differ amongst Aichi Targets. Patterns of shortfalls will also vary across groups of Parties: megadiverse countries likely assign more value and, therefore, priority and legislative power to biodiversity, though in recent history, they may have suffered greater biodiversity losses due to limited financial and technical capacity on top of differing national priorities. Finally, there is evidence that multilateral environmental agreements such as the CBD do not always have sufficient leverage over other entities, such as the private sector or other government ministries outside of the environment, to enact substantial change in them^27,35^.

### Data collection

Using a combination of text-mining techniques and manual extraction, we created two databases of planned (NBSAPs) and implemented (Sixth National Reports) actions. We joined these datasets together to allow for comparison between Parties that have reported variable coverage of their successes. We then used this database to connect commitments directly to the Aichi Targets with a mixture of links at the planned, implementation, and target levels, analysing differences in the reported effectiveness of implementation between them. Finally, we compared groups of Parties’ reports to determine how biodiversity progress in the so-called “like-minded megadiverse countries” has differed from the rest of the world, both overall and across the Aichi Targets.

We took a subset of Parties’ national planning and reporting data that were available from the CBD Clearing-House Mechanism with a temporal cut-off of 31st January 2020. This Clearing-House Mechanism (https://www.cbd.int/chm/) is a digital platform to which Parties to the CBD can upload planning and reporting documents, and where these documents are hosted and retrievable by third party entities. Three criteria in succession were used to select Parties. First, the Parties in this subset needed a Sixth National Report and a connected NBSAP; this narrowed the subset to 83 Parties (Supplementary Table 1). Second, we required Parties that have submitted a Sixth National Report to also have submitted an NBSAP in the period after the Aichi Targets were adopted, 2011-onwards (64 Parties met criteria 1 and 2). Third, we required that Parties had itemised, hierarchical action plans in their NBSAPs. Our final subset comprised 58 Parties, covering a range of regions and Human Development Index (HDI) categories (Table 1).

**Table 1:** The distribution of Parties in our subset of NBSAPs and Sixth National Reports. Regional groups come from the UN Regions, and the Human Development Index groups come from the World Bank

UN region	Parties in a subset (and % of all countries in the region)
Africa	17 (31%)
Asia-Pacific	16 (30%)
Eastern Europe	7 (30%)
Latin America and the Caribbean	13 (39%)
Western Europe and Others	5 (18%)

Human development index category	Parties in subset (and % of all countries in category)
High	39 (34%)
Medium	10 (27%)
Low	9 (25%)

### Data extraction

Some action plans are in lists of activities, such as those from Japan, the Republic of Korea, and Belgium. These could be extracted by hand into a database, with indicators, deadlines, and higher-level categories recorded manually in separate fields. Other action plans were tabular—in various types of hierarchy, with different definitions for the levels of commitment, including action, activity, target, objective, strategic objective, operational objective, and theme. Here, a hierarchical methodology was used to extract data from tabular NBSAPs, which could include hundreds of individual commitments per country. In the case of Ecuador, where a database of actions and indicators was provided in an accessible format alongside its NBSAP, its columns were converted to the harmonised format, and no further action was taken. The PDF file format commonly used for published NBSAPs does not preserve tables in an accessible, transparent format, so we used the Tabula software^31^ and manual cleaning to extract these and convert them into the harmonised format. Once the raw data from NBSAP files had been collected, we set definitions for data collection to ensure harmony between Parties (Table 2).

**Table 2:** Definitions used when harmonising commitment data from NBSAPs

Term	Definition
Target	The highest-level commitment with the exception of where commitments were in line with their relevant Aichi Target and another grouping category existed above—usually named “Thematic Area” or something similar. These were kept in country-level datasets but omitted from the harmonised structure, as many action plans did not include them.
Action	The commitments at the lowest hierarchical level each country’s NBSAP provided were assigned as “Actions”. These are used as the basic unit of commitment in our analysis. The only exception to this is where indicators have been used as the lowest hierarchical level of planning, in which case they were pooled to the action level. For a commitment to be labelled as an action, we stipulated a “commitment to act”: some action needed to be mentioned, so commitments simply highlighting the importance of doing something or warning of consequences should an action not be taken were not included.
Objective	The next-up hierarchical level of commitment from actions—these are the most commonly referenced levels of commitment in the reporting phase (National Reports).
Indicator	The “end state” of an action is usually a measurable quantity associated with the commitment. Whether this was an indicator of implementation (e.g., “the legislation is signed”) or of effect (e.g., “the population of species X rises to 300 breeding pairs”) varied amongst Parties and commitments.
Deadline	The date by which an action was to have been completed. A comparison between national commitments with a deadline before and after 2020 would potentially mislabel commitments to be carried out after 2020 as unsuccessful/ absent if they were not mentioned in the National Report, so we included the individual deadline for each action (if recorded) in order to filter out commitments that were not relevant to the Sixth National Reports. Where no date was referenced in the text of the NBSAP, the date for a related objective or target was used instead. If no date was available, we used the deadline for the NBSAP itself (usually 2020).

Our subset of 58 Parties yielded a total of 7931 commitments, with the average Party presenting 95 commitments in its NBSAP. All commitments have an action associated; almost all (>99%) have an indicator (though not necessarily at action-level detail); almost all (>99%) have an objective (though in the simplest hierarchical structures by our definitions, this was also a target).

Parties are encouraged to submit the Sixth National Reports to the CBD through the CBD Clearing-House Mechanism, a repository for the files that hosts them all together and is searchable by categories, including country, information type, and keyword. The submission process for a Sixth National Report using this mechanism is somewhat systematic, with set values for many of the fields filled in. It is also presented, once published, in a more unified format, allowing for mechanical scraping of the information. Using a defined form, the PDF files containing National Report data follow reliable rules which can be exploited to tabulate the data. Tabular, tidy data could, therefore be extracted automatically from these documents using parsing algorithms and regular expressions. We used the ‘tidytext’ R package^37^ to extract the measures taken by each Party, recording information on the objective the measures contributed to, the body of the measure, any associated national targets, the reported effectiveness of the measure, and any obstacles noted by the Party that impeded the measure’s progress.

Some Parties, such as Botswana and Chile, linked each of their actions directly to “measures taken” in their reporting system. These are already harmonised to the greatest extent amongst the Parties in our subset. Others used higher-level classifications to report, leaving the information on individual actions within blocks of text. Fuzzy matching, longest common subsequence & longest common substring and regular expressions were used to identify passages in National Reports in which actions and their outcomes have been assessed. Where no mention of a commitment was detected, each measure reported in the National Report was manually checked for a mention of the original commitment. The documents were presented in a range of languages. We generated machine translations of key fields for interpretation, but comparisons and links were analysed in the original language in all but one case (where Andorra’s NBSAP was given in Spanish, and its Sixth National Report was in French). Nonetheless, we tested for the effect of reporting language on apparent implementation effectiveness and found no significant difference between NBSAPs written in English, Spanish, or French (all NBSAPs were available in one of these languages).

Finally, there were a number of Parties (<5) where their reporting structure, and even planning structures, had changed between NBSAP and National Report publication. These were read and tabulated manually. The unified data on links between commitments and the National Reports across all levels of planning and reporting were collated into a new field in the database. A total of 10,651 links were made across all national commitment portfolios. We classified measures that were mentioned in the NBSAPs but had no mention in the National Reports as “omissions” rather than certain failures or non-started actions (Table 2, Supplementary Fig. 3). Together, these methods created a unified dataset detailing the existence of records of progress towards national commitments to biodiversity from initial action-planning, through an assessment of measures taken, to the eventual contribution of those measures to the achievement of national targets, with two stages of progress assessment at the measure and target level. Alongside the initial data from the NBSAPs, this can provide insight into the trajectory of national commitment success and eventually progress towards achieving the Aichi Targets.

### Matching national commitments to Aichi Targets

We made use of several ways in which Parties reported the connections between their commitments and the Aichi Targets. First, at the NBSAP level, in their action plans, many Parties linked objectives and actions directly to Aichi Targets by including a column in their tabular action plan. Secondly, some Parties did not link their initial commitments to the Aichi Targets but linked the “measures taken” in their Sixth National Reports to their relevant Aichi Targets, so it was possible to work backwards to link the commitment to an Aichi Target. Finally, some Parties did not link their commitments to the Aichi Targets but to their own national targets; in this case, we drew links between those national targets and the Aichi Targets using the ‘National Targets’ section of the CBD data site if they were not clearly linked one-to-one with Aichi Targets. In all, we were able to link 56% of commitments in the database to an Aichi Target using these methods. For the purposes of comparing commitment implementation specifically between Aichi Targets (Figs. 1 and 2), highly connected commitments (more than 4 linked Aichi Targets) were not included to minimise multiple counting and draw distinctions between Targets.

### Data analysis

Once collected and classified, the proportion of a Party’s initial commitments linked to each Aichi Target with reportedly effective delivery was tested against 2 factors: The Human Development Index category of the Party^38^ and whether it was made by a “megadiverse” country as defined by the “Like-Minded Megadiverse Countries”^20^, or not. We used the Kruskal-Wallace test (‘kruskal.test’, base R^39^) and a post-hoc Dunn test where appropriate (‘dunn_test’, rstatix package^40^) using commitments’ reported success as a binary dependent variable to the Aichi Targets where commitments were considered individually (Fig. 2). Where national proportions of commitments have been compared on a by-Party basis (as in Fig. 3), We used a one-way ANOVA function ‘aov’ in base R^39^ to identify if any differences existed between groups.

### Supplementary information

SI_FINAL

## Data Availability

This analysis generated a dataset of national commitments to biodiversity, complete with our linking attempts, in relation to Sixth National Reports and National Targets. We have made the full database accessible to facilitate further research and analysis (10.6084/m9.figshare.24943371).
