# Supplementary material for: National commitments to Aichi Targets and their implications for monitoring the Kunming-Montreal Global Biodiversity Framework
Source: NPJ Biodivers. 2024 Apr 3;3:6. doi: 10.1038/s44185-024-00039-5 (PMC11332214; doi:10.1038/s44185-024-00039-5)
Supplement: Supplementary file 1 — SI_FINAL [file 44185_2024_39_MOESM1_ESM.docx]

### Supplementary Table 1

Supplementary Table 1. The Parties to the Convention on Biological Diversity with a 6^th^ National Report as of the study period, and the criteria each of them met to be included in this study. Parties are presented by the ISO3 code relevant to their country, and the Parties included in the study are presented first.

| **Country (ISO3)** | **Date of most recent report** | **Included in study** | **Condition 1** | **Condition 2** | **LMMC** |
| --- | --- | --- | --- | --- | --- |
| DZA | 30/12/2018 | TRUE | TRUE | TRUE | NO |
| AND | 20/12/2018 | TRUE | TRUE | TRUE | NO |
| BLR | 29/12/2018 | TRUE | TRUE | TRUE | NO |
| BEL | 17/05/2019 | TRUE | TRUE | TRUE | NO |
| BLZ | 14/05/2019 | TRUE | TRUE | TRUE | NO |
| BTN | 31/12/2018 | TRUE | TRUE | TRUE | NO |
| BIH | 05/07/2019 | TRUE | TRUE | TRUE | NO |
| BWA | 31/05/2019 | TRUE | TRUE | TRUE | NO |
| BFA | 29/03/2019 | TRUE | TRUE | TRUE | NO |
| CMR | 30/06/2019 | TRUE | TRUE | TRUE | NO |
| CHL | 07/06/2019 | TRUE | TRUE | TRUE | NO |
| CHN | 09/01/2019 | TRUE | TRUE | TRUE | YES |
| HRV | 31/10/2019 | TRUE | TRUE | TRUE | NO |
| DOM | 19/07/2019 | TRUE | TRUE | TRUE | NO |
| ECU | 28/12/2018 | TRUE | TRUE | TRUE | YES |
| EGY | 30/06/2019 | TRUE | TRUE | TRUE | NO |
| EST | 04/04/2019 | TRUE | TRUE | TRUE | NO |
| FIN | 25/04/2019 | TRUE | TRUE | TRUE | NO |
| GMB | 15/03/2019 | TRUE | TRUE | TRUE | NO |
| GTM | 15/10/2020 | TRUE | TRUE | TRUE | YES |
| IND | 29/12/2018 | TRUE | TRUE | TRUE | YES |
| IDN | 14/10/2019 | TRUE | TRUE | TRUE | YES |
| ITA | 12/04/2019 | TRUE | TRUE | TRUE | NO |
| JPN | 25/12/2018 | TRUE | TRUE | TRUE | NO |
| JOR | 09/04/2019 | TRUE | TRUE | TRUE | NO |
| KGZ | 22/04/2019 | TRUE | TRUE | TRUE | NO |
| LBN | 12/06/2019 | TRUE | TRUE | TRUE | NO |
| MDV | 20/07/2019 | TRUE | TRUE | TRUE | NO |
| MLI | 01/03/2019 | TRUE | TRUE | TRUE | NO |
| MDA | 02/01/2019 | TRUE | TRUE | TRUE | NO |
| MAR | 17/05/2019 | TRUE | TRUE | TRUE | NO |
| MMR | 30/12/2018 | TRUE | TRUE | TRUE | NO |
| NPL | 05/01/2019 | TRUE | TRUE | TRUE | NO |
| NER | 15/12/2018 | TRUE | TRUE | TRUE | NO |
| NGA | 27/12/2018 | TRUE | TRUE | TRUE | NO |
| PAN | 22/10/2019 | TRUE | TRUE | TRUE | NO |
| PRY | 26/12/2018 | TRUE | TRUE | TRUE | NO |
| PER | 28/12/2018 | TRUE | TRUE | TRUE | YES |
| POL | 29/05/2019 | TRUE | TRUE | TRUE | NO |
| QAT | 03/11/2019 | TRUE | TRUE | TRUE | NO |
| KOR | 28/03/2019 | TRUE | TRUE | TRUE | NO |
| KNA | 24/10/2022 | TRUE | TRUE | TRUE | NO |
| VCT | 06/06/2019 | TRUE | TRUE | TRUE | NO |
| SEN | 05/12/2019 | TRUE | TRUE | TRUE | NO |
| SVK | 10/07/2020 | TRUE | TRUE | TRUE | NO |
| ZAF | 21/12/2018 | TRUE | TRUE | TRUE | YES |
| ESP | 06/02/2019 | TRUE | TRUE | TRUE | NO |
| LKA | 17/07/2020 | TRUE | TRUE | TRUE | NO |
| SDN | 31/12/2018 | TRUE | TRUE | TRUE | NO |
| TZA | 05/05/2019 | TRUE | TRUE | TRUE | NO |
| THA | 10/03/2020 | TRUE | TRUE | TRUE | NO |
| TGO | 31/12/2018 | TRUE | TRUE | TRUE | NO |
| TTO | 02/08/2019 | TRUE | TRUE | TRUE | NO |
| TUN | 14/03/2019 | TRUE | TRUE | TRUE | NO |
| URY | 07/01/2019 | TRUE | TRUE | TRUE | NO |
| VUT | N/A | TRUE | TRUE | TRUE | NO |
| VEN | 04/07/2019 | TRUE | TRUE | TRUE | YES |
| ZMB | 28/02/2019 | TRUE | TRUE | TRUE | NO |
| SUR | 09/01/2020 | FALSE | TRUE | FALSE | NO |
| ARE | 23/12/2019 | FALSE | TRUE | TRUE | NO |
| PLW | 18/12/2019 | FALSE | TRUE | FALSE | NO |
| GRC | 10/12/2019 | FALSE | TRUE | TRUE | NO |
| SOM | 03/12/2019 | FALSE | TRUE | TRUE | NO |
| LVA | 01/10/2019 | FALSE | TRUE | FALSE | NO |
| LCA | 21/08/2019 | FALSE | TRUE | FALSE | NO |
| TJK | 24/08/2019 | FALSE | TRUE | FALSE | NO |
| LIE | 30/07/2019 | FALSE | TRUE | TRUE | NO |
| NLD | 17/07/2019 | FALSE | TRUE | TRUE | NO |
| SAU | 30/06/2019 | FALSE | TRUE | FALSE | NO |
| CRI | 03/06/2019 | FALSE | TRUE | FALSE | YES |
| ETH | 14/04/2019 | FALSE | TRUE | FALSE | YES |
| PHL | 02/04/2019 | FALSE | TRUE | FALSE | YES |
| SWE | 29/03/2019 | FALSE | TRUE | FALSE | NO |
| GBR | 01/05/2019 | FALSE | TRUE | FALSE | NO |
| KAZ | 27/02/2019 | FALSE | TRUE | FALSE | NO |
| ATG | 07/08/2019 | FALSE | TRUE | TRUE | NO |
| CIV | 24/12/2019 | FALSE | TRUE | FALSE | NO |
| MEX | 02/07/2019 | FALSE | TRUE | FALSE | YES |
| CZE | 16/05/2019 | FALSE | TRUE | FALSE | NO |
| CAN | 10/06/2019 | FALSE | TRUE | FALSE | NO |
| LUX | 21/12/2018 | FALSE | TRUE | FALSE | NO |
| NOR | 21/12/2018 | FALSE | TRUE | FALSE | NO |
| CHE | 19/12/2018 | FALSE | TRUE | FALSE | NO |

### Supplementary Figure 1


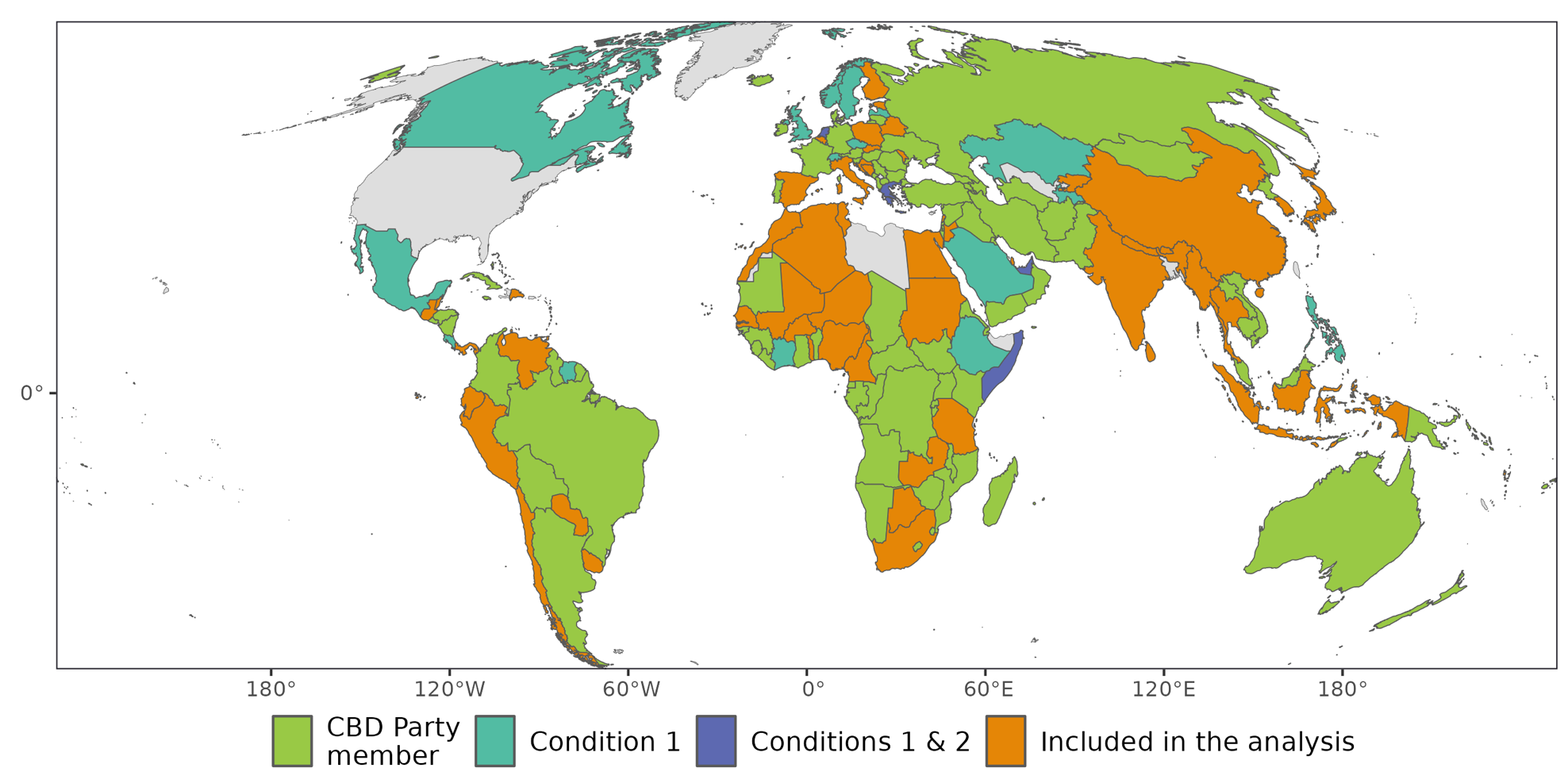


Supplementary Figure 1. The distribution of Parties included in this study globally. Countries marked in green are CBD party members, but did not meet Criterion 1 or 2 for this analysis. Countries marked in light blue had a NBSAP and connected National Report, but did not have an NBSAP linked to the Aichi Targets; territories marked in dark blue had a linked NBSAP and 6^th^ National Report that were connected to the Aichi Targets, but did not have an itemised action plan suitable for analysis in their NBSAP. The remaining orange countries were included in the analysis. The designations employed and the presentation of material on the above map do not imply the expression of any opinion whatsoever on the part of the Secretariat of the United Nations concerning the legal status of any country, territory, city or area or of its authorities, or concerning the delimitation of its frontiers or boundaries.

### Supplementary Figure 2


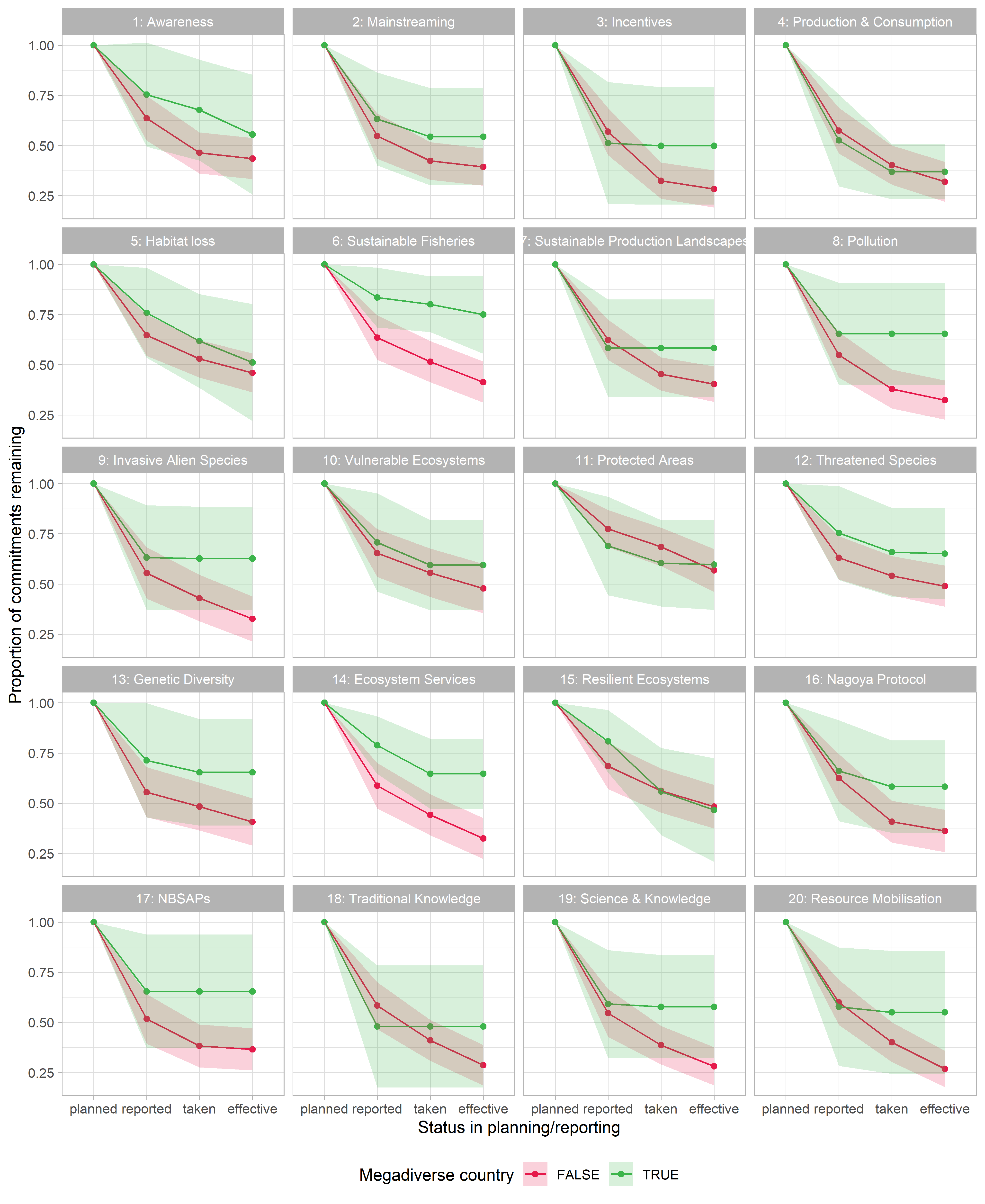


Supplementary Figure 2. Differences between countries within and outside the group of “Like-Minded Megadiverse Countries”^16^ – which include Bolivia, Brazil, China, Colombia, Costa Rica, Democratic Republic of the Congo, Ecuador, Ethiopia, Guatemala, India, Indonesia, Iran, Kenya, Madagascar, Malaysia, Mexico, Peru, Philippines, South Africa, and Venezuela. The countries from this group in our subset of Parties are marked in Table S1.

### Supplementary Figure 3


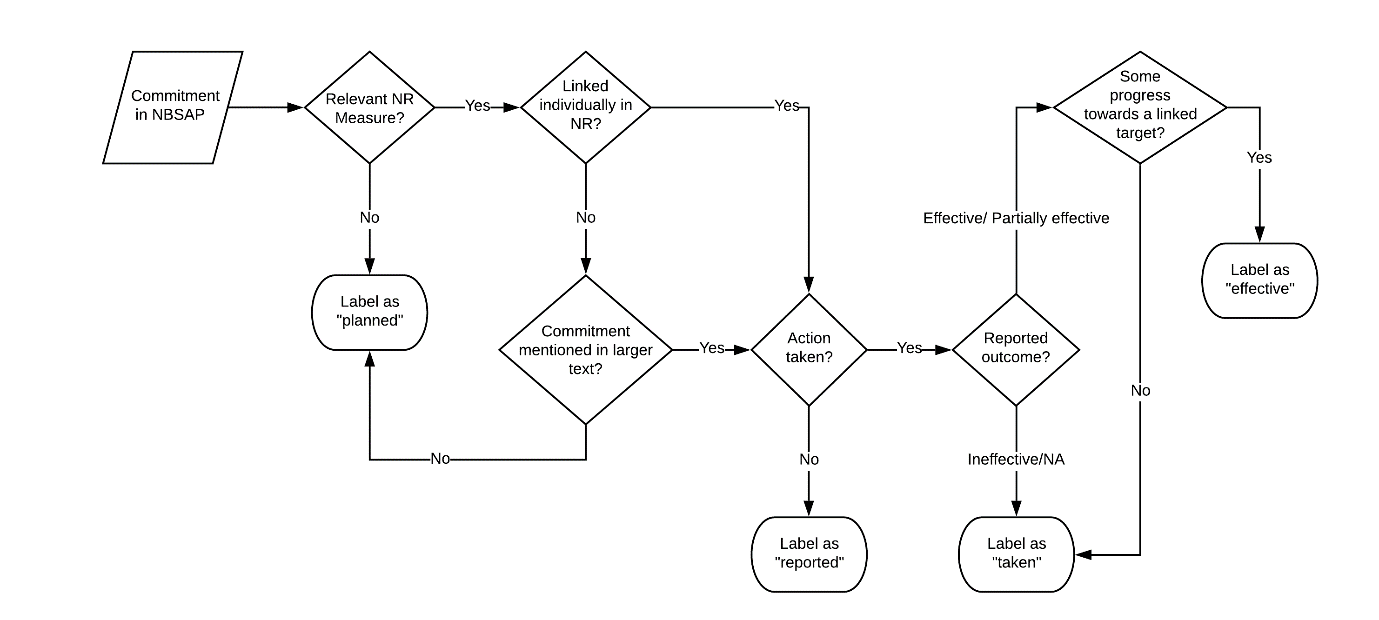


*Supplementary Figure 3. The process used to classify commitments into one of four categories. Here, NBSAP refers to a country’s National Biodiversity Strategy and Action Plan, and NR refers specifically to the Sixth National Reports we used in this study.*
